# Oncological outcomes of direct *versus* delayed surgery after neoadjuvant short-course radiotherapy for rectal cancer: national cohort study

**DOI:** 10.1093/bjsopen/zrag146

**Published:** 2026-10-13

**Authors:** Najia Azhar, Marie-Louise Lydrup, Pamela Buchwald

**Affiliations:** Department of Clinical Sciences Malmö, Lund University, Malmö, Sweden; Department of Surgery, Skåne University Hospital, Malmö, Sweden; Department of Clinical Sciences Malmö, Lund University, Malmö, Sweden; Department of Surgery, Skåne University Hospital, Malmö, Sweden; Department of Clinical Sciences Malmö, Lund University, Malmö, Sweden; Department of Surgery, Skåne University Hospital, Malmö, Sweden

**Keywords:** study implementation, real-world data, clinical practice

## Abstract

**Background:**

After the Stockholm III trial, a trend towards delayed surgery after neoadjuvant short-course radiotherapy (5 × 5 Gy) for rectal cancer has emerged in Sweden, driven by the lower short-term complication rates and similar oncological outcomes. This study aimed to examine long-term oncological outcomes after neoadjuvant short-course radiotherapy, comparing direct *versus* delayed surgery for rectal cancer in a real-world setting.

**Methods:**

A registry-based retrospective national cohort study was conducted using prospectively collected data from the Swedish Colorectal Cancer Registry. Patients with rectal cancer receiving neoadjuvant neoadjuvant short-course radiotherapy with surgery between 2016 and 2020 were analysed for oncological outcomes (local recurrence, distant metastasis) and overall survival at 3 years, comparing direct surgery, defined as surgery within 7 days after neoadjuvant short-course radiotherapy, and delayed surgery, defined as surgery after 28–90 days. The rate of patients who underwent surgery within the delayed time frame of the Stockholm III trial was also recorded.

**Results:**

A total of 2080 patients were identified, of whom 1208 (58.1%) underwent direct surgery and 872 had delayed surgery. Of note, only 288 patients underwent resection 28–56 days after neoadjuvant short-course radiotherapy (within the time frame of the Stockholm III trial). Although the pathological complete response rate was higher in the delayed-surgery group compared with the direct-surgery group (6.5% *versus* 0.4%), 3-year local recurrence and distant metastasis rates were, respectively, 1.0% (12 patients) and 6.8% (82) in the direct-surgery group, and 3.4% (30) and 11.9% (104) in the delayed-surgery group. The adjusted hazard ratios for local recurrence and distant metastasis comparing delayed surgery with direct surgery were 3.60 (95 per cent confidence interval 1.75 to 7.39) and 2.17 (1.58 to 2.96), respectively, whereas the adjusted hazard ratio for overall survival was 1.37 (1.06 to 1.77), favouring the direct-surgery group.

**Conclusion:**

In a real-world setting, the results indicate that few patients in the delayed-surgery group had surgery within the stipulated time frame. Considering this, delayed surgery was associated with a higher risk of local recurrence, distant metastasis, and reduced overall survival.

## Teaser

This was a national cohort study comparing direct *versus* delayed surgery for rectal cancer after neoadjuvant short-course radiotherapy in a real-world setting. The results show better oncological outcomes after direct surgery on evaluation after 3 years and, importantly, that most patients undergo surgery beyond the time frame stipulated in the protocol for delayed surgery.

## Introduction

Neoadjuvant radiotherapy reduces the risk of local recurrence in rectal cancer, but overall survival benefits have been debated, as well as treatment-related morbidity, resulting in a debate over selective use^1–5^. However, the use of neoadjuvant radiotherapy lacks worldwide consensus, and national guidelines vary^6,7^. The timing of surgery after neoadjuvant treatment has been debated recently. Standard approaches include short-course radiotherapy (SCRT) at 5 × 5 Gy and direct surgery (within 7 days), or long-course radiotherapy at 50 Gy, often with concomitant radiosensitizing chemotherapy, with surgery after 4–8 weeks, depending on staging at multidisciplinary conferences. The Swedish Stockholm III trial investigated the timing of surgery, comparing SCRT with direct surgery and delayed surgery (4–8 weeks after SCRT completion) and with long-course chemoradiotherapy (50 Gy). The trial concluded that delayed surgery was associated with fewer postoperative complications, not graded in terms of severity, but similar oncological outcomes. Subgroup analysis revealed higher tumour regression grades with delayed surgery after SCRT, potentially translating into improved survival^8,9^, findings supported by a Polish study^10^.

A Turkish study^11^ also reported overall survival benefits. The results have led a change in clinical practice in Sweden since 2016 towards SCRT with delayed surgery, although some regions still practice standard treatment with direct surgery and use delayed surgery in selected patients^12^. Such variation offers a unique opportunity to evaluate this change, how the implementation of study results translates into a real-world setting, and whether oncological safety can be preserved outside a study protocol. Previous research has disclosed several challenges during implementation, in which personal opinions, clinical practice, and the ease of the intervention can affect guideline adherence^13^. The latest European Society for Medical Oncology guidelines^14^ propose that both direct and delayed surgery can be used after SCRT.

This study aimed to examine oncological outcomes, comparing SCRT with direct or delayed surgery outside a study protocol, and to assess how changes in clinical practice translate to a real-world setting.

## Methods

A registry-based retrospective national cohort study using prospectively collected data from the Swedish Colorectal Cancer Registry (SCRCR) was conducted. This registry has been validated previously^15,16^. Inclusion criteria were all patients with a rectal cancer diagnosis without distant metastases, who received neoadjuvant SCRT and had surgery between 2016 and 2020.

The time frames used were the same as in the Stockholm III trial for direct surgery; for delayed surgery, the window was extended to 90 days rather than 56 days, reflecting a shift towards later surgery in the delayed-surgery group. The authors assumed that patients undergoing surgery within 4 weeks beyond the original upper limit were intended for the delayed-surgery group.

The study was approved by the Swedish Ethical Review Authority (2022-06216-01).

### Outcomes of interest

The primary outcome was local recurrence rate at 3 years. Secondary outcomes were distant metastasis rate and overall survival. Patients were compared by resection time frame; thus, comparisons were made between direct surgery (< 7 days after SCRT) and delayed surgery (28–90 days after SCRT).

The rate of patients who underwent surgery within the delayed time frame of the Stockholm III trial (28–56 days after SCRT) was also recorded.

A subgroup analysis was undertaken using the original Stockholm III time frames and clinical (c) T3 *versus* cT4 rectal cancers.

### Statistical analysis

Median (interquartile range, i.q.r.) and mean with standard deviation were calculated for continuous variables. Categorical data were compared using Pearson's χ^2^ test.

For overall survival, local recurrence, and distant metastasis, Cox proportional hazard models were employed, with results presented as hazard ratios (HRs) with 95% confidence intervals. Kaplan–Meier graphs were used to present overall survival, local recurrence, and distant metastasis by timing of surgery. All models were adjusted for age, sex, American Society of Anesthesiologists (ASA) grade, operation type, adjuvant chemotherapy, and cT and N categories. *P* < 0.050 was considered statistically significant. Statistical analysis was undertaken in R version 4.3.3 (R Project for Statistical Computing, Vienna, Austria).

## Results

### Study cohort

A total of 3971 patients were identified who underwent surgery for rectal cancer between 2016 and 2020, and 1565 were excluded as they received neoadjuvant treatment other than SCRT or had metastatic disease at diagnosis (*Fig. 1*). Of the remainder, 13 patients were excluded because radiotherapy dates were missing, leaving 2375. Thirty-three patients underwent surgery 11–27 days after completion of radiotherapy and so could not be classified as having either direct or delayed surgery, and were excluded. A further 262 patients were excluded because they underwent surgery > 90 days after completion of radiotherapy; they were assumed to belong to another category of patients, possibly included in a watch-and-wait protocol. The remaining 2080 patients treated with SCRT were divided into those who had direct surgery (< 7 days after completion of radiotherapy; 1208) and those had delayed surgery (28–90 days after completion of radiotherapy; 872).

**Figure 1:**
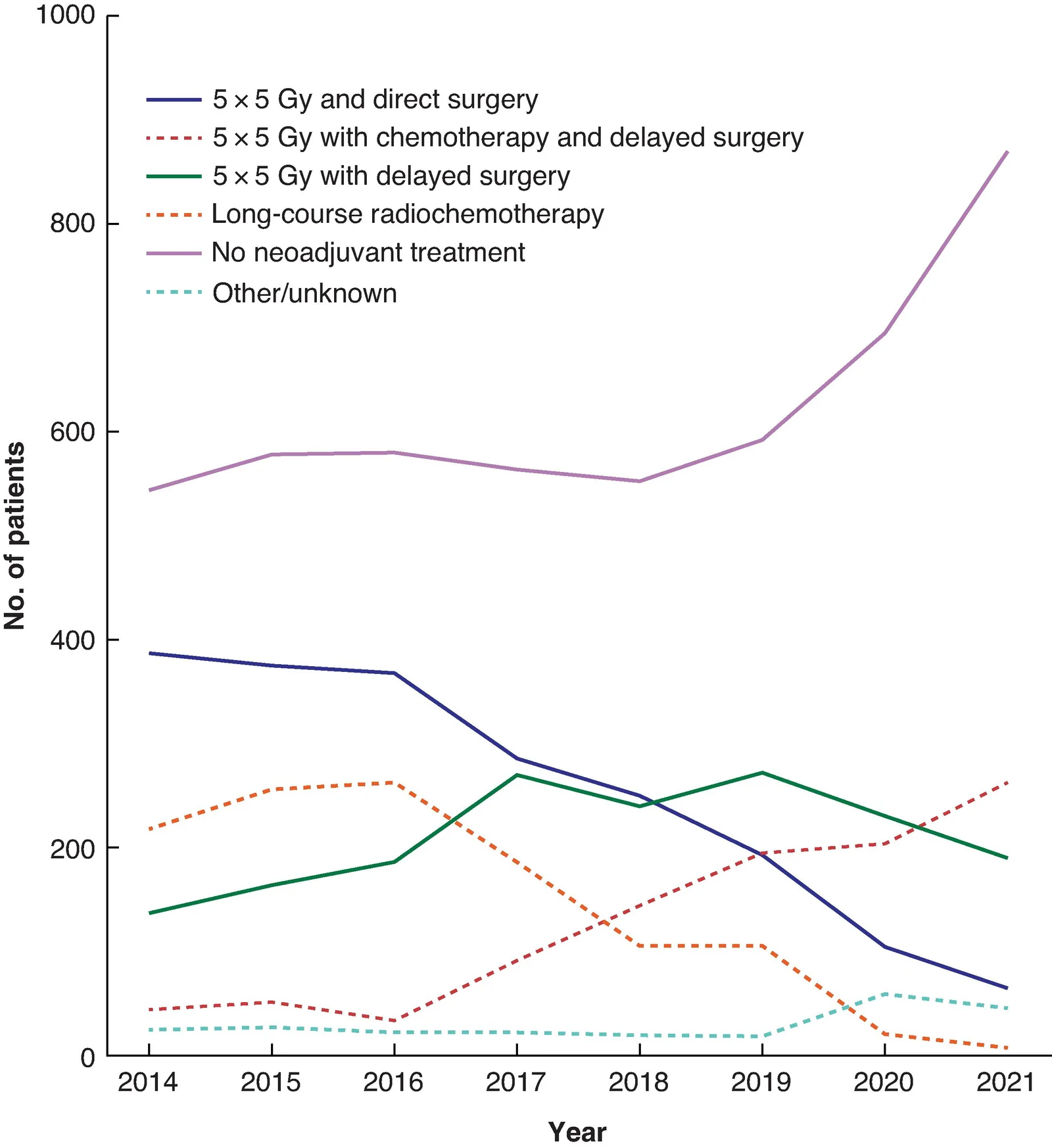
Neoadjuvant treatment in Sweden from 2014–2021 Data from Swedish Colorectal Cancer Registry^12^.

Of note, using the original Stockholm III trial time frame would have resulted in inclusion of only 288 patients in the delayed-surgery group, compared with 872 when the extended interval was applied.

### Study population

Data from the SCRCR confirmed the trend towards use of the regimen comprising 5 × 5 Gy with chemotherapy and delayed surgery (*Fig. 2*).

**Figure 2:**
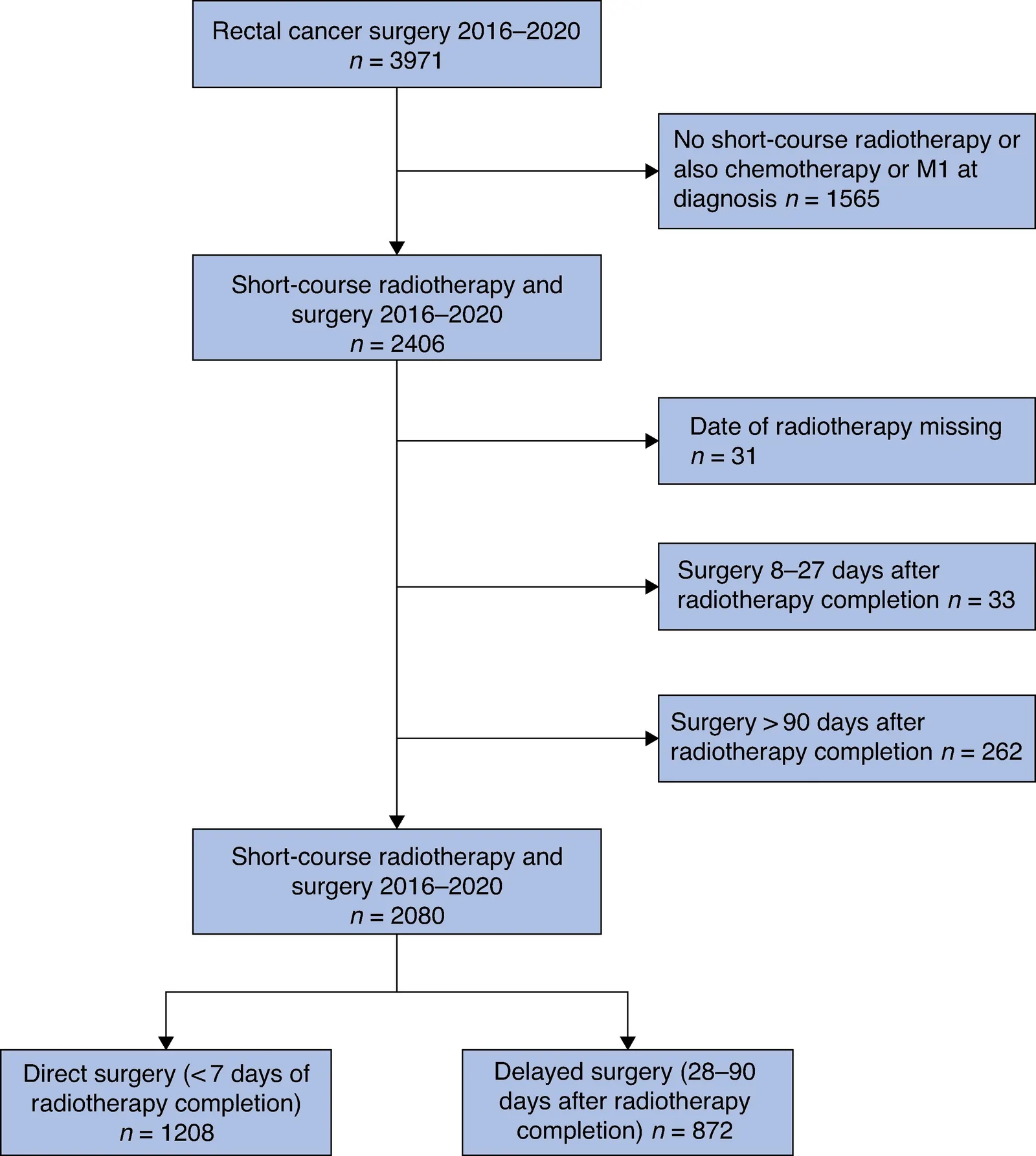
Study flow chart

Clinical characteristics are summarized in *Table 1*. Median age was 70 (i.q.r. 62–75) and 73 (66–79) years in the direct-surgery and delayed-surgery groups, respectively, and 38% of participants were women in both groups (*Table 1*). The median interval to surgery was 3 (i.q.r. 2–3) and 65 (54–74) days, respectively. The most common procedure was abdominoperineal resection, followed by anterior resection and then Hartmann's operation. Anterior resection was significantly more common in the direct-surgery group and Hartmann’s operation in the delayed-surgery group. The groups were otherwise similar, but patients with an ASA grade of III–IV and those with cT4 tumours were more common in the delayed-surgery group. Adjuvant chemotherapy was more commonly used in the direct-surgery group (30% *versus* 11%).

**Table 1:** Patient characteristics by timing of surgery

	Overall (*n* = 2080)	Direct surgery (*n* = 1208)	Delayed surgery (*n* = 872)	P*
Age (years), median (range)	71 (27–94)	70 (31–89)	73 (27–94)	
**Sex**				0.981
Male	1290 (62%)	750 (62%)	540 (62%)	
Female	790 (38%)	458 (38%)	332 (38%)	
**ASA grade**				< 0.001
I	341 (17%)	251 (21%)	90 (11%)	
II	1160 (57%)	705 (59%)	455 (53%)	
III	506 (25%)	218 (18%)	288 (34%)	
IV	29 (1%)	8 (1%)	21 (2%)	
V	3 (0%)	3 (0%)	0 (0%)	
Missing	41 (2%)	23 (2%)	18 (2%)	
BMI (kg/m^2^), median (range)	25.7 (12.5–54.0)	25.8 (16.9–46.1)	25.6 (12.5–54.0)	
Missing	641 (31%)	419 (34%)	222 (25%)	
**Type of operation**				< 0.001
Anterior resection	831 (40%)	516 (43%)	315 (36%)	
Abdominoperineal resection	972 (47%)	567 (47%)	405 (46%)	
Hartmann's operation	248 (12%)	111 (9%)	137 (16%)	
Other	23 (1%)	12 (1%)	14 (2%)	
Missing	3	2	1	
**Clinical tumour category**				< 0.001
cT1–T2	485 (23%)	322 (27%)	163 (19%)	
cT3	1305 (63%)	770 (64%)	535 (61%)	
cT4	264 (13%)	100 (8%)	164 (19%)	
cTx	23 (1%)	15 (1%)	8 (1%)	
Missing	3	1	2	
**Clinical node status**				0.243
cN0	690 (33%)	417 (35%)	273 (31%)	
cN1–N2	1372 (66%)	778 (64%)	535 (61%)	
cNx	15 (1%)	12 (1%)	3 (0%)	
Missing	3	1	2	
Adjuvant chemotherapy	455 (22%)	358 (30%)	97 (11%)	< 0.001
Pathological complete response	62 (3%)	5 (0%)	57(7%)	< 0.001
Pathological N1–N2	804 (39%)	507 (42%)	297 (34%)	< 0.001
Harvested lymph nodes < 12	195 (9%)	108 (9%)	87 (10%)	0.422
R0 resection	2000 (96%)	1172 (97%)	828 (95%)	0.016

Values are *n* (%) unless otherwise stated. ASA, American Society of Anesthesiologists; BMI, body mass index. *Pearson's χ^2^ test.

The pathological complete response (pCR) rate was higher in the delayed-surgery group (6.5%, 57 patients) compared with the direct-surgery group (0.4%, 5). The percentage of patients with < 12 harvested lymph nodes was 9% in the direct-surgery group and 10% in the delayed-surgery group. The rate of node positivity was 42% in the direct-surgery group and 34% in the delayed-surgery group. R0 resection rates were 97% and 95%, respectively.

### Study outcomes

At 3-year follow-up, the local recurrence rate was 2.1% overall (42 patients), 1.1% (12) in the direct-surgery group and 3.6% (30) in the delayed-surgery group. Distant metastasis and mortality rates were higher in the delayed-surgery group: 6.8% (82 patients) *versus* 11.9% (104) and 10.8% (131) *versus* 18.9% (165) (*Table 2*).

**Table 2:** Oncological outcomes at 3-year follow-up

	Overall (*n* = 2080)	Direct surgery (*n* = 1208)	Delayed surgery (*n* = 872)
**Local recurrence**	42 (2.1%)	12 (1.1%)	30 (3.6)
Missing	123 (5.8%)	92 (7.5%)	31 (3.5%)
**Distant metastasis**	186 (9.0%)	82 (6.8%)	104 (11.9%)
Missing	272 (13.0%)	172 (14.2%)	99 (11.4%)
**Death**	296 (14.0%)	131 (10.8%)	165 (18.9%)
Missing	2 (0%)	2 (0.2%)	0 (0%)

Values are *n* (%.)

Unadjusted Cox logistic regression analysis revealed statistically significant differences between direct and delayed surgery for local recurrence, distant metastasis, and overall survival (*Table 3*). After adjusting for age, sex, ASA grade, operation type, adjuvant chemotherapy, cT and N status, there were still statistically significant differences between the groups in local recurrence, distant metastasis, and overall survival, favouring the direct-surgery group (*Table 3*).

**Table 3:** Results of unadjusted and adjusted Cox regression analysis for delayed surgery (*versus* direct surgery)

	Unadjusted analysis			Adjusted analysis		
	*n*	Hazard ratio	*P*	*n*	Hazard ratio	*P*
Local recurrence	1957	3.49 (1.79, 6.81)	< 0.001	1894	3.60 (1.75, 7.39)	< 0.001
Distant metastases	1782	1.83 (1.37, 2.44)	< 0.001	1728	2.17 (1.58, 2.96)	< 0.001
Overall survival	2078	1.83 (1.37, 2.44)	< 0.001	2008	1.37 (1.06, 1.77)	0.016

Values in parentheses are 95% confidence intervals. Direct surgery is the reference category (hazard ratio 1.00). *Adjusted for age, sex, American Society of Anesthesiologists grade, operation type, adjuvant chemotherapy, tumour category, and node status.

A subgroup analysis was undertaken comparing direct *versus* delayed surgery using the Stockholm III time frames (direct: 1–7 days after SCRT; delayed: 28–56 days after SCRT). The two groups had similar baseline characteristics regarding age and sex, but ASA grade I was more common in the direct-surgery group and grade III–IV in the delayed-surgery group (*Table S1*). The most common surgical procedure was anterior resection in both groups (59%), but Hartman’s operation was again more common in the delayed-surgery group than the direct-surgery group (27% *versus* 18%; *P* < 0.001) as were cT4 tumours (17% *versus* 8%). Adjuvant chemotherapy was also more commonly used in the direct-surgery group in this cohort (30% *versus* 12%; *P* < 0.001) (*Table S1*).

At 3-year follow-up, the total local recurrence rate was 1.6% (22 patients), 1.0% (12) in the direct-surgery group and 3.6% (10) in the delayed-surgery group. Distant metastasis and mortality rates were higher in the delayed-surgery group: 6.8% (82 patients) *versus* 9.0% (26) and 10.8% (131) *versus* 17.7% (51) (*Table S2*).

After adjusting for age, sex, ASA grade, operation type, adjuvant chemotherapy, cT category and N status, adjusted Cox logistic regression analysis revealed statistically significant differences between direct and delayed surgery for local recurrence (HR 3.69, 95% confidence interval 1.49 to 9.13; *P* = 0.006) and distant metastasis (HR 1.83, 1.15 to 2.91; *P* = 0.015) favouring the direct-surgery group (*Table S3*).

Another subgroup analysis was conducted comparing outcomes of patients with cT3 compared with cT4 tumours. The adjusted Cox regression analysis revealed a higher local recurrence rate among those with cT3 tumours in the delayed-surgery group (HR 3.04, 1.21 to 7.65; *P* = 0.013); however, for those with cT4 tumours, the results were not significant (HR 2.51, 0.50 to 12.17; *P* = 0.25) (*Tables S4* and *S5*).

## Discussion

The timing of surgery after SCRT has been debated. Direct surgery requires rigorous surgical organization, with scheduling of the surgical procedure in close proximity to completion of radiotherapy. If oncological results were similar with delayed surgery, this could significantly alleviate operational planning, and, unsurprisingly, it has emerged as an attractive option in many colorectal departments in Sweden. This change in clinical practice has, however, not yet been evaluated outside a study protocol, and many factors could contribute to difficulties in implementing study results in clinical practice^13^. The present national cohort study, using real-life data, has indicated a difference in oncological outcomes favouring direct surgery, with the strong reservation that very few patients in the delayed-surgery group had surgery within the time frame originally stipulated. Some departments have not implemented delayed surgery for all patients, but reserve it for elderly and more frail patients. Still, the results remain significant, even after adjusting for these variables, and are in accordance with those of a large Canadian study^17^ that showed better overall survival if surgery was conducted within 3 weeks after SCRT.

In Sweden, the shift towards delayed surgery occurred largely because of the results of the Stockholm III trial, in which delayed surgery was performed 28–56 days after completion of SCRT. Interestingly, in the present study, most patients had surgery within a later time frame than was proposed in the Stockholm III trial, as the median time to surgery was 65 days, a whole week later than the upper limit in that trial^8^. Possible explanations include surgical planning logistics and the challenges of adhering to strict timelines outside a study protocol. Delays may also have been related to patient-specific factors such as co-morbidities, treatment-related toxicity, and patient preference, which were identified as important causes of delay in the Canadian cohort^17^. It cannot be ruled out that this influenced outcomes. This also highlights the challenges in translating study results into clinical practice. In the present study, a subgroup analysis with strict adherence to the Stockholm III time frame was conducted. The results for local recurrence and distant metastasis were similar to those of the main analysis, but there was no statistically significant difference between the groups in overall survival (*Tables S1–S3*).

The strengths of this study include its large national cohort, prospectively collected from a validated registry, which mirrors everyday clinical practice. However, several limitations should be acknowledged. As a registry-based study, data quality cannot be more reliable than that of the registry. The SCRCR data have been validated^15^, with special attention to oncological outcomes^18^, but still individual outcomes cannot be assessed and there are some missing data and/or also a risk of misclassification errors. Re-evaluation in the delayed-surgery group is also unknown in the registry; therefore, there are no data available on disease progression, which could contribute to selection bias that cannot be adjusted for. From the authors’ original cohort, 33 patients were excluded as they had surgery within a time frame that could not be called either direct or delayed, and 262 patients had surgery later than 90 days after completion of SCRT. Reasons why so many patients had surgery later than proposed in the protocol can only be speculated on; they may include patient frailty, surgical scheduling logistics, having had disease progression, or being included in a watch-and-wait protocol.

Previous studies^9,17^ have reported a higher pCR rate with delayed surgery. The present findings concur with these results, and it is accepted that a pCR is associated with better oncological outcomes and disease-free survival^19,20^, which may be a reason to consider delayed surgery. Such results were not recorded in this cohort and reasons for this need more consideration.

The use of SCRT and direct surgery has been decreasing in Sweden since the emergence of SCRT and concomitant chemotherapy as well as surgery without any neoadjuvant treatment from 2016 onwards^12^. Internationally, this regimen has never been as common as in the Nordic countries, which may limit the scope of this study. In 2020, SCRT with chemotherapy or SCRT with delay were the most commonly used neoadjuvant regimens^12^, as variants of the RAPIDO study protocol have emerged. RAPIDO like neoadjuvant treatment have changed what is most commonly used now. Even though the results of the present study affect fewer patients, they are still valuable findings in the assessment of how study results are implemented in general clinical practice, how changes occur even before the results are published, and for the importance of continually evaluating changes in clinical practice.

To conclude, this national cohort study comparing direct *versus* delayed surgery for rectal cancer after SCRT has shown better oncological outcomes after direct surgery at 3-year evaluation. Furthermore, most patients had surgery beyond the time frame stipulated in the protocol that induced the shift towards delayed surgery. The reasons for this should be investigated and evaluated, in case they influence oncological outcomes.

## Supplementary Material

zrag146_Supplementary_Data

## Data Availability

Data will be available upon reasonable request.
