# Supplementary material for: Oncological outcomes of direct versus delayed surgery after neoadjuvant short-course radiotherapy for rectal cancer: national cohort study
Source: BJS Open. 2026 Oct 13;10(5):zrag146. doi: 10.1093/bjsopen/zrag146 (PMC13660688; doi:10.1093/bjsopen/zrag146)
Supplement: Supplementary file 1 [file zrag146_supplementary_data.docx]

**Oncological outcomes after changes in clinical practice of direct vs delayed surgery after neoadjuvant short-course radiotherapy for rectal cancer: a national cohort study**

Najia Azhar ^1,2^, Marie-Louise Lydrup ^1,2^, Pamela Buchwald ^1,2^

1. Department of Clinical Sciences Malmö, Lund University, Malmö Sweden
2. Department of Surgery, Skåne University Hospital, Malmö, Sweden

**Corresponding author**

Najia Azhar

Jan Waldenströms gata 38

20585 Malmö, Sweden

**Email:** [najia.azhar@med.lu.se](mailto:najia.azhar@med.lu.se)

**Orcid:** **0000-0001-6709-4441**

**Supplementary Materials - Index**

| **Supplementary Figures and Tables** |  |
| --- | --- |
| **Table S1**. Baseline characteristics for direct surgery <7days and delayed surgery 28-56 days after SCRT. | *page. 2* |
| **Table S2.** Oncological Outcomes for direct surgery <7days and delayed surgery 28-56 days after SCRT.  **Table S3**. Adjusted Cox regression analysis for timing of surgery for direct surgery <7days and delayed surgery 28-56 days after SCRT.  **Table S4**. Adjusted Cox regression analysis for timing of surgery for cT3 cancers. Direct surgery <7 days and delayed surgery 28-90 days after SCRT.  **Table S5**. Adjusted Cox regression analysis for timing of surgery for cT4 cancers. Direct surgery <7 days and delayed surgery 28-90 days after SCRT. | *page. 3*  *page.3*  *page.3*  *page.4* |
|  |  |
|  |  |

**Supplementary Figures and Tables**

**Table S1**. Baseline characteristics for direct surgery <7days and delayed surgery 28-56 days after SCRT.

| **Variable** | **Overall  N = 1,496^1^** | **Direct surgery  N = 1,208^1^** | **Delayed surgery  N = 288^1^** |
| --- | --- | --- | --- |
| Age | 70 [31, 89] | 70 [31, 89] | 72 [41, 88] |
| Sex |  |  |  |
| Male | 934 (62%) | 750 (62%) | 184 (64%) |
| Female | 562 (38%) | 458 (38%) | 104 (36%) |
| ASA class |  |  |  |
| I | 282 (19%) | 251 (21%) | 31 (11%) |
| II | 870 (59%) | 705 (59%) | 165 (59%) |
| III | 295 (20%) | 218 (18%) | 77 (27%) |
| IV | 16 (1.1%) | 8 (0.7%) | 8 (2.8%) |
| V | 3 (0.2%) | 3 (0.3%) | 0 (0%) |
| Missing | 30 | 23 | 7 |
| BMI | 25.8 [16.9, 46.1] | 25.8 [16.9, 46.1] | 25.9 [18.2, 42.4] |
| Missing | 498 | 402 | 96 |
| Type of operation |  |  |  |
| Anterior resection | 632 (43%) | 516 (43%) | 116 (41%) |
| APR | 694 (47%) | 567 (47%) | 127 (45%) |
| Hartmann´s operation | 150 (10%) | 111 (9.3%) | 39 (14%) |
| Missing | 20 | 14 | 6 |
| T stage |  |  |  |
| T1-2 | 381 (26%) | 322 (27%) | 59 (21%) |
| T3 | 946 (63%) | 770 (64%) | 176 (61%) |
| T4 | 150 (10%) | 100 (8.3%) | 50 (17%) |
| Tx | 17 (1.1%) | 15 (1.2%) | 2 (0.7%) |
| Missing | 2 | 1 | 1 |
| N stage |  |  |  |
| N0 | 514 (34%) | 417 (35%) | 97 (34%) |
| N1-2 | 968 (65%) | 778 (64%) | 190 (66%) |
| Nx | 12 (0.8%) | 12 (1.0%) | 0 (0%) |
| Missing | 2 | 1 | 1 |
| Adjuvant chemotherapy | 392 (26%) | 358 (30%) | 34 (12%) |
| ^1^Median [range] for continuous; n (%) for categorical | | | |

**Table S2.** Oncological outcomes for direct surgery <7days and delayed surgery 28-56 days after SCRT.

| **Variable** | **Overall  N = 1,496^1^** | **Direct surgery  N = 1,208^1^** | **Delayed surgery  N = 288^1^** |
| --- | --- | --- | --- |
| Mortality | 182 (12%) | 131 (11%) | 51 (18%) |
| Missing | 2 | 2 | 0 |
| Local recurrence | 22 (1.6%) | 12 (1.1%) | 10 (3.6%) |
| Missing | 105 | 92 | 13 |
| Distant metastasis | 108 (8.5%) | 82 (7.9%) | 26 (11%) |
| Missing | 218 | 172 | 46 |
| Major complications | 30 (2.0%) | 27 (2.2%) | 3 (1.0%) |
| ^1^Median [range] for continuous; n (%) for categorical | | | |

**Table S3**. Adjusted Cox regression analysis for timing of surgery for direct surgery <7days and delayed surgery 28-56 days after SCRT. Direct surgery is reference=1

| Variable | N | HR (95% CI) | p |
| --- | --- | --- | --- |
| Local recurrence | 1345 | 3.69 (1.49-9.13) | 0.006 |
| Distant metastases | 1225 | 1.83 (1.15- 2.91) | 0.015 |
| Overall survival | 1443 | 1.37 (0.96-1.95) | 0.087 |

* Adjusted for age, sex, ASA class, operation type, adjuvant chemotherapy, T-and N stage.

**Table S4**. Adjusted Cox regression analysis for timing of surgery for cT3 cancers. Direct surgery <7 days and delayed surgery 28-90 days after SCRT.

Direct surgery is reference=1

| Variable | N | HR (95% CI) | p |
| --- | --- | --- | --- |
| Local recurrence | 1198 | 3.04 (1.21-7.65) | 0.013 |
| Distant metastases | 1095 | 2.13 (1.45- 3.15) | <0.001 |
| Overall survival | 1265 | 1.31(0.95-1.81) | 0.093 |

* Adjusted for age, sex, ASA class, operation type, adjuvant chemotherapy, and N stage

**Table S5**. Adjusted Cox regression analysis for timing of surgery for cT4 cancers. Direct surgery <7 days and delayed surgery 28-90 days after SCRT.

Direct surgery is reference=1

| Variable | N | HR (95% CI) | p |
| --- | --- | --- | --- |
| Local recurrence | 236 | 2.51 (0.50-12.17) | 0.253 |
| Distant metastases | 201 | 3.42 (1.04-11.2) | 0.029 |
| Overall survival | 253 | 1.91 (0.96-3.82) | 0.056 |

* Adjusted for age, sex, ASA class, operation type, adjuvant chemotherapy, and N stage
